# Lineage 2 West Nile Virus as Cause of Fatal Neurologic Disease in Horses, South Africa

**DOI:** 10.3201/eid1506.081515

**Published:** 2009-06

**Authors:** Marietjie Venter, Stacey Human, Dewald Zaayman, Gertruida H. Gerdes, June Williams, Johan Steyl, Patricia A. Leman, Janusz Tadeusz Paweska, Hildegard Setzkorn, Gavin Rous, Sue Murray, Rissa Parker, Cynthia Donnellan, Robert Swanepoel

**Affiliations:** University of Pretoria, Pretoria, South Africa (M. Venter, S. Human, D. Zaayman, J. Williams, J. Steyl, C. Donnellan); National Health Laboratory Services, Pretoria (M. Venter); Onderstepoort Veterinary Research Institute, Pretoria (G.H. Gerdes); National Institute for Communicable Diseases, Johannesburg, South Africa (P. Leman, J.T. Paweska, R. Swanepoel); Chartwell Equine Clinic, Midrand, South Africa (H. Setzkorn); Karoo Veterinary Clinic, Colesburg, South Africa (G. Rous); Witbos Clinic, Midrand (S. Murray); Glen Austin Equine Clinic, Midrand (R. Parker)

**Keywords:** Zoonoses, West Nile Virus, encephalitis, horses, Africa, viruses, research

## Abstract

Serologic evidence suggests that West Nile virus (WNV) is widely distributed in horses in southern Africa. However, because few neurologic cases have been reported, endemic lineage 2 strains were postulated to be nonpathogenic in horses. Recent evidence suggests that highly neuroinvasive lineage 2 strains exist in humans and mice. To determine whether neurologic cases are being missed in South Africa, we tested 80 serum or brain specimens from horses with unexplained fever (n = 48) and/or neurologic signs (n = 32) for WNV. From March 2007 through June 2008, using reverse transcription–PCR (RT-PCR) and immunoglobulin (Ig) M ELISA, we found WNV RNA or IgM in 7/32 horses with acute neurologic disease; 5 horses died or were euthanized. In 5/7 horses, no other pathogen was detected. DNA sequencing for all 5 RT-PCR–positive cases showed the virus belonged to lineage 2. WNV lineage 2 may cause neurologic disease in horses in South Africa.

## Abstract 2

Lineage 2 WNV may be missed as a cause of neurologic infections in horses and humans in this region.

West Nile virus (WNV), a mosquito-born flavivirus of the family *Flaviviridae*, is widely distributed throughout Africa, the Middle East, Asia, parts of Europe, Australia, North and South America, and the Caribbean. The WNV transmission cycle involves birds as vertebrate hosts and ornithophilic mosquitoes as maintenance vectors (*1*). Isolates of WNV fall into 2 major genetic lineages: lineage 1 is found in North America, North Africa, Europe, and Australia; lineage 2 strains are endemic to southern Africa and Madagascar (*2*,*3*). Recently, additional lineages in central and eastern Europe (lineages 3 and 4) (*4*,*5*) and India (lineage 5) have been reported (*6*).

Humans and horses are incidental hosts for WNV (*7*). Although most infections are benign, ≈20% of infected persons have fever, rash, arthralgia, and myalgia, and for ≈1% of these, severe disease, including meningoencephalitis, encephalitis, and polio-like flaccid paralysis, may develop. Rare cases result in hepatitis, myocarditis, pancreatitis (*8*), and death (*1*). Signs in horses are ataxia, weakness, recumbency, and muscle fasciculation (*9*–*11*). Seroepidemiologic studies suggest that asymptomatic infections frequently occur in horses (*12*,*13*), but neurologic infections result in a high case-fatality rate (30%–40%) (*14*). In 2002 the largest outbreak of WNV encephalomyelitis in horses was recorded in the United States; 15,257 cases were reported from 40 states (*11*). This outbreak was followed in 2003 by the largest outbreak in humans in the Northern Hemisphere (9,832 cases) (*1*). The number of cases among horses was greatly reduced after the introduction of an inactivated vaccine for animals (*10*,*15*,*16*).

In the Karoo, a semidesert region in South Africa, in 1974, WNV caused one of the largest outbreaks ever recorded in humans, affecting tens of thousands of people. During this outbreak thousands of persons visited their local clinicians; however, no cases of neurologic disease were reported. In the 1980s, an epizootic involving WNV and Sindbis virus occurred in the Witwatersrand area of the Gauteng Province in South Africa; this epizootic affected hundreds of persons (*17*). Since then, the number of confirmed human cases has been ≈5–15 per year, although only a proportion of cases are subjected to laboratory investigation. In South Africa, severe disease has been recognized, including fatal hepatitis and several nonfatal encephalitis cases in humans as well as deaths in ostrich chicks, a foal, and a dog (*2*,*18*). Recently, a lineage 2 strain was isolated from encephalitic birds in central Europe, which suggests that lineage 2 strains can spread outside their known geographic range and may cause severe disease in birds in non–WNV-endemic countries (*19*).

A recent serologic survey of thoroughbred horses has confirmed that WNV is widely distributed throughout South Africa; 11% of yearlings seroconverted over 1 year and up to 75% of their dams had been exposed (*13*). This study led to the postulation that endemic lineage 2 WNV strains were not a cause of neuroinvasive disease in horses because none of these horses had shown any clinical signs. Three seronegative horses inoculated with a recent WNV lineage 2 strain (SPU381/00) isolated from a person with benign disease did not develop clinical signs (*13*). However, the strain used in these experiments was subsequently shown to be of low neuroinvasiveness in mice, compared with certain other South African strains (*20*). Subclinical cases are also frequently reported in horses in the United States (*12*). Experimental infection of 12 horses with the highly neuroinvasive NY99 strain resulted in neuroinvasive disease in only 1 animal; the remaining animals all seroconverted, but clinical disease did not develop and virus could not be isolated from their organs (*21*).

Comparison of South African and North American strains of WNV has shown that differences in neuroinvasiveness are associated with specific genotypes, not with lineage, and that highly neuroinvasive strains exist in lineages 1 and 2 (*20*,*22*). To determine whether equine cases of WNV are being missed in South Africa, for 16 months we investigated horses with pyrexia or unexplained neurologic signs.

## Materials and Methods

### Clinical Cases

From March 2007 through June 2008, serum and/or postmortem brain specimens were collected from horses in South Africa with acute fever or neurologic disease; cases were detected by passive surveillance. Specimens were sent to the Department of Medical Virology, University of Pretoria, by the main veterinary diagnostic facilities in South Africa (Onderstepoort Veterinary Institute and the University of Pretoria Faculty of Veterinary Science, Onderstepoort) and by a group of private equine veterinarians from Gauteng and the Northern Cape provinces, who were invited to submit samples from horses with suspected cases. Specimens from horses with fatal and severe neurologic cases mostly came from the University of Pretoria (Pathology Department, Faculty of Veterinary Sciences) and the Onderstepoort Veterinary Institute; most were from horses with neurologic signs for which no alternative diagnoses were made. Specimens from horses with fever were collected by the private veterinarians around Gauteng from horses with less severe disease. Cases that resembled African horse sickness, i.e., pulmonary or cardiac disease, were not included.

### Reverse Transcription–PCR Screening and DNA Sequencing

RNA was extracted with an RNeasy kit (QIAGEN, Hilden, Germany) according to the manufacturer’s recommendations. A nested real-time reverse transcription–PCR (RT-PCR) specific for the WNV NS5 gene that distinguishes between lineages 1 and 2 with WNV-specific FRET probes on the basis of dissociation curve analysis was used to screen specimens (*23*). A product of 214 bp could be seen on agarose gel. All positive RT-PCR products were confirmed by sequencing of the NS5 region (genome positions 9091–9191) and analyzed on an ABI 3130 sequencer as recommended by the supplier (Applied Biosystems, Foster City, CA, USA). RT-PCR amplification and sequence analysis of a 255-bp region of the E-protein (genome positions 1402–1656) was conducted as described before (*2*,*24*).

### Phylogenetic Analysis

Sequences were aligned by using ClustalX version 1.83 (http://bips.u-strasbg.fr/fr/Documentation/ClustalX) with the multiple-sequence alignment option. Maximum-likelihood trees were generated by using PHYML (*25*). Bootstrap statistics for 1,000 replicates were calculated by neighbor-joining analysis with a maximum composite likelihood model and a gamma parameter of 2, using MEGA version 4 (*26*). Distances between sequences were calculated by using MEGA version 4 (*26*) with the P-distance analysis option.

### Serologic Testing

Horse serum samples were tested for flavivirus-specific antibodies first by hemagglutination inhibition (HI) assay and next by a WNV-specific immunoglobulin (Ig) M-capture ELISA on HI-positive specimens as described in (*27*). IgM-positive specimens were confirmed by neutralization assays as described below.

### IgM-Capture ELISA

The IgM-capture ELISA was conducted as described in (*28*) with virus-specific modifications. In brief, 100 µL/well of goat antihorse IgM µ-chain (Kirkegaard and Perry Laboratories, Inc., Gaithersburg, MD, USA) diluted 1:500 in phosphate-buffered saline (PBS) without magnesium and calcium, pH 7.4, was adsorbed onto ELISA plates (MaxiSorp, Nunc, Denmark) overnight in a humidity chamber at 4°C. Plates were washed 3× with 0.1% Tween-PBS; the same washing procedure followed each subsequent stage of the assay. Plates were blocked with 200 µL/well of 10% skim milk (Merck, Darmstadt, Germany) in PBS and incubated at 37°C for 1 h. After washing, duplicate volumes of 100 µL of each test and control serum diluted 1:400 in 2% skim milk in PBS (diluting buffer) were added to wells in rows A–D:1–12 and to corresponding wells in rows E–G:1–12 and incubated at 37°C for 1 h. After washing, 100 µL/well WNV antigen and mock antigen, diluted 1:400 in diluting buffer, was added to rows A–D:1–12 and E–G:1–12, respectively. After incubation at 37°C for 1 h and washing, 100 µL/well of mouse anti-WNV antibody diluted 1:1,000 was added to each well and incubated at 37°C for 1 h. Production, inactivation, preservation, and safety testing of WNV antigen (strain H442/58), mock antigen, and hyperimmune mouse anti-H442/58 serum were conducted as described (*29*). After washing, 100 µL/well goat antimouse IgG (H + L chain) HRPO-conjugate (Zymed Laboratories, Inc, San Francisco, CA, USA) diluted 1:2,000 was added to each well and incubated at 37°C for 1 h. Plates were washed, and 100 µL/well ABTS (2,2′-azino diethyl-benzothiazoline-sulfonic acid) peroxidase substrate (Kirkegaard and Perry Laboratories, Inc.) was added to each well and the plate was incubated in the dark for 30 min at room temperature (22°–25°C). The stop reagent, 1% sodium dodecyl sulfate, was added, and optical densities (ODs) were measured at 405 nm. Specific activity of each serum sample (net OD) was calculated by subtracting the nonspecific OD in wells with mock antigen from the specific OD in wells with virus antigen. A threshold value for interpretation of results was determined as mean plus 3 standard deviations of duplicate net OD readings for negative control serum.

### HI Assay

The HI assay was conducted as described previously (*27*), except that the sucrose-acetone-extracted H442/58 strain of WNV derived from mouse brain tissue (produced as described for the IgM-capture ELISA above) was used as an antigen. A serum sample was considered seropositive if it had a titer >1.3, equivalent to a serum dilution >1:20.

### Serum Neutralization Test

The serum microneutralization procedure using African green monkey kidney (Vero cells) was conducted as described previously (*30*), except that the SPU 93/01 isolate of WNV recovered in South Africa was used as a source of antigen. The titer was expressed as the reciprocal serum dilution that inhibited 100% of viral cytopathic effect. A serum sample was considered positive when it had a virus neutralization titer >1.0, equivalent to a serum dilution >1:10.

### Tests for Differential Diagnoses

African horse sickness virus (AHSV), equine encephalosis virus (EEV), and equine herpesviruses (EHV) 1 and 4 were identified by using viral culture and antigen detection assays and/or complement fixation tests (*31*) on serum samples and using RT-PCR to detect AHSV and EHV (*32*). Rabies virus infections were identified by fluorescent antigen detection tests on brain tissue (*33*). Immunoperoxidase staining for EEV, EHV, AHSV, and flavivirus was performed, according to the method adapted from (*34*), on histopathologic sections of brain, spinal cord, spleen, liver, and lung after postmortem investigations.

### Virus Culture

All specimens were inoculated onto Vero cell monolayers (18 hours old) in 25 cm^2^ tissue culture flasks supplemented with Eagle Minimum Essential Medium containing 2% fetal bovine serum, 100 IU/mL penicillin, 100 μg/mL streptomycin, and 1mg/mL l-glutamine (GIBCO BRL, Invitrogen, Carlsbad, CA, USA). Inoculated cultures were microscopically observed for cytopathic effects for 10 days.

## Results

### Screening of Specimens

A total of 80 serum or brain specimens from horses with unexplained fever (n = 48) and/or neurologic symptoms (n = 32) were tested for WNV over the 16-month period. Most specimens from horses with neurologic signs came from Onderstepoort Veterinary Institute and the Department of Medical Virology, University of Pretoria; horses were from across the country; the specimens from horses with fever were mostly from horses with less severe disease in Gauteng.

WNV infection was identified for 7 (21.8%) of 32 acute neurologic cases (Table 1). For 5 cases, acute WNV infection could be confirmed by the presence of RNA through RT-PCR (4 brain specimens, 1 serum sample) as well as virus isolation from 1 brain specimen. Two HI-positive cases could be confirmed as probable recent WNV infections by IgM ELISA and WNV-specific antibodies, which were confirmed by neutralization assays. None of the horses with fever had WNV, although EEV was isolated from several. Affected horses ranged from 4 months to 19 years and were thoroughbreds, Arabians, Lipizzaners, Welsh ponies, warmbloods, and mixed breeds. Cases were identified in Gauteng, the Northern Cape, and North-Western provinces and occurred in April 2007 and from March through June 2008. Of the 7 WNV-infected horses, 5 died or were euthanized for humane reasons (Table 2).

**Table 1:** Viral diagnostic findings, West Nile virus–infected horses, South Africa*

Case no.	Date sample received	Location	Specimen	Final diagnosis	Results of tests for other viruses
SAE12/07	2007 Apr 23	Johannesburg, Sandton, Gauteng	Plasma	WNV IgM+	AHSV–, EEV–
SAE89/08	2008 Mar 3	Colesburg North Cape	Plasma	WNV IgM+	AHSV–, EEV–
HS101/08	2008 Apr 15	Tiegerpoort Pretoria, Gauteng	Brain	WNV PCR+, DNA sequencing L2, virus isolae WNV PCR+	Rabies–, AHSV–, EEV–, EHV–, flavivirus antigen in lumbar spinal cord section and in some gray matter axons (Figure 1)
M123/08	2008 May 8	Midrand, Gauteng	Brain	WNV PCR+, DNA sequencing L2	AHSV–, EEV–, EHV–
HS125/08	2008 May 26	Pretoria, Hammans-kraal, Gauteng	Brain	WNV PCR+, DNA sequencing L2	AHSV from spleen PCR+; AHSV from lung IHC+, lymph node IHC–, liver IHC–
SAE126/08	2008 Mar 7	Midrand, Gauteng	Brain	WNV PCR+, DNA sequencing L2	AHSV RT-PCR+, AHSV type 7; IHC EEV–, AHSV+, AHSV IHC+ (lung liver, heart)
SAE134/09	2008 Jul 18	Potchef-stroom, North-Western Province	Serum	WNV PCR+, DNA sequencing L2	AHSV–, EEV–, EHV-1 weak sero+

*WNV, West Nile virus, Ig, immunoglobuliln; AHSV, African horse sickness virus; EEV, equine encephalosis virus; EHV, equine herpesvirus; L2, lineage 2; –, negative; +, positive; RT-PCR, reverse transcription–PCR; IHC, immunohistochemistry.

**Table 2:** Clinical findings, West Nile virus–infected horses, South Africa*

Case no.	Age	Clinical findings	Outcome	Postmortem findings	Histopathologic findings
SAE12/07	5 y	Neurologic signs, hind and fore limb ataxia, pupil miosis, head held to left	Survived		
SAE89/08	1 y	Neurologic signs, hind and forelimb ataxia, fever, complete paralysis, anorexia, hepatitis	Died (euthanized after week of fluid therapy)	Postmortem not done	
HS101/08	8 y	Neurologic signs, severe ataxia especially hind limb, seizures and chewing, froth from mouth, fever, recumbency, paralysis	Died	Marked generalized subcutaneous edema involving trunk and proximal forelimbs, edema (periaortic, coronary grooves, neck, and hind quarters), partial pulmonary collapse, foam-filled trachea, mild serosanguinous hydrothorax, moderate hydropericardium, subpleural petechiae and ecchymoses, epicardial grooves and at bases of the mitral valves	Lesions in gray matter and meninges of lumbar spinal cord (pericentral canal gliosis, edema, gray matter gliosis with occasional neuronal degeneration or death), mild perivascular cuffing with mononuclear cells and scattered neutrophils, moderate vascular congestion, occasional perivascular petechiae, mild leptomeningitis (mostly round cells) and occasional mild spinal ganglioneuritis, segmental mural necrosis of the dural blood vessels and neutrophil invasion, less-marked lesions in rest of spinal cord and white matter of midbrain
M123/08	4 mo	Fever, neurologic signs, paralysis (Schiff-Sherington sign), rectal prolapse	Died	Severe perirenal and intermuscular edema, severe diffuse interlobular lung edema and mild serous hydropericardium	Marked white matter lesions in peripheral lateral and ventromedial spinal cord white matter, mild perivascular round cell cuffing in midbrain, patchy spongiosis and gliosis in brain and cerebellum white matter
HS125/08	8 mo	Neurologic signs, ataxia, mild colic, swollen head and neck	Died (shot by owner)	Severe lung edema and congestion, moderate serous hydropericardium, intermuscular edema	Subtle lesions on brain and cerebellum sections (e.g., gliosis and spongiosis in white matter and vascular leukostasis)
SAE126/08	6 y	Neurologic signs, head hanging, sick for week	Died suddenly		Spinal cord lesions
SAE134/09	19 y	Neurologic signs, partial blindness, hyperexcitability, seizures	Survived		

*AHSV, African horse sickness virus; IHC, immunohistochemistry.

### Co-infections

For 5 horses, 3 of which had fatal infections, no virus other than WNV was identified. Two horses that died (8 months of age and 6 years of age) had co-infections with AHSV. For both horses, WNV was detected by RT-PCR in the brain and AHSV was detected in the spleen or lungs but not in the brain. The 6-year-old horse had documented records of up-to-date AHSV vaccinations. All cases were negative by immunoperoxidase staining, virus isolation, and/or PCR for EHV and EEV.

### Clinical Description of WNV

All confirmed WNV infections were identified in the group of horses with neurologic signs (Tables 1,2). Five horses with WNV died or had to be euthanized after becoming paralyzed. Signs included ataxia in all cases (7/7), weak hindlimbs and/or forelimbs and paresis (4/7), complete paralysis (2/7), seizures (2/7), chewing (1/7), partial blindness (2/7), jaundice and/or hepatitis (2/7), and miosis of the pupils (1/7). One horse (HS101/08) (Table 2) was recumbent from quadriplegia and displayed limb paddling, teeth grinding, and muscle twitching; signs progressed over 3 days to those similar to rabies, i.e., chewing fits, seizures, and coma before death, but fluorescent antigen detection results for rabies were negative. Fever was intermittent and not reported for all horses. The 2 WNV-infected horses that survived showed clinical signs for ≈21 days and had to be rested for several months, but each recovered fully.

**Figure 1:**
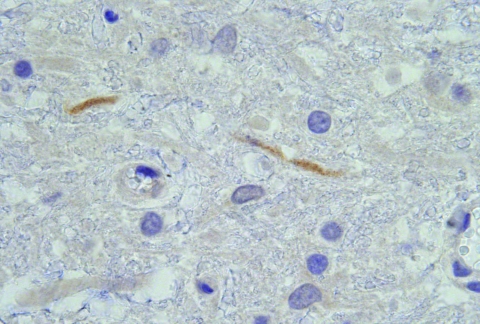
Histopathologic section of 2 lumbar spine gray matter dendrites that stained immunohistochemically positive with flavivirus antiserum on postmortem tissue of horse HS101/08. Magnification ×1,000.

Postmortem investigations were performed on 4 horses (HS125/08, SAE 126/08, M123/08, and HS101/08). All 4 had positive WNV results by real-time RT-PCR of brain tissue, and a WNV isolate was obtained from the brain of HS101/08. Virus isolation attempts on frozen specimens from the other 3 horses were not successful. AHSV was also isolated from the lungs and spleen of SAE126/08 and HS125/08. Immunohistochemical staining of organs, to identify AHSV, EEV, and EHV, confirmed the presence of AHSV in SAE126/08, but results were negative for all other horses. Detailed postmortem findings for the 2 horses that had no complications (HS101/08 and M123/08) are shown in Table 2. Immunohistochemical staining for flaviviruses demonstrated antigen in the lumbar spinal cord and in some gray matter axons of HS101/08 (Figure 1).

### Sequence Confirmation and Phylogenetic Analysis

**Figure 2:**
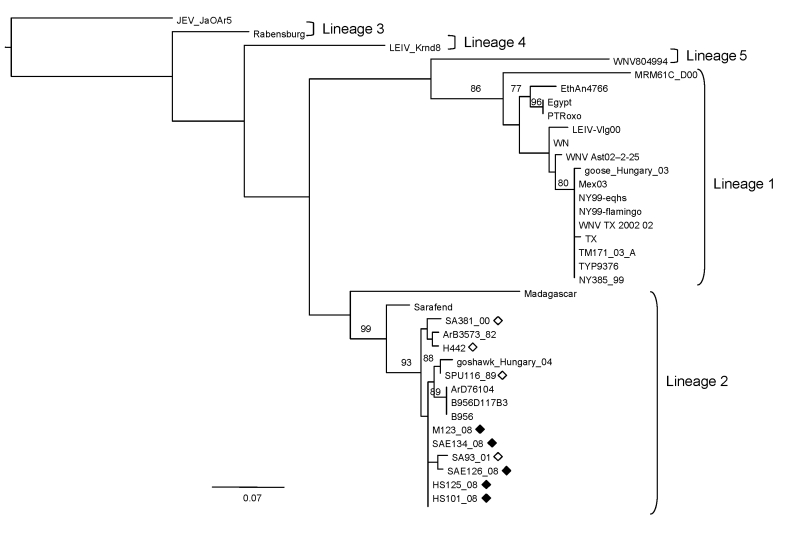
Maximum-likelihood comparison of the partial NS5 gene of West Nile virus (WNV) strains identified in horses in South Africa in 2008 with representative sequences of other WNV lineages. Bootstrap statistics are shown on the branches; only values >70% are included. Scale bar indicates 0.07 nt changes. Japanese encephalitis virus (JEV) was used as an outgroup. Black diamonds, WNV strains identified in horses in South Africa in the present study; white diamonds, WNV strains isolated from humans in South Africa in previous years. WNV strains and accession numbers and origin: HS123_08, FJ464376, South Africa; HS125_08, FJ464377, South Africa; HS101_08, FJ464378, South Africa; SAE126_08, FJ464379, South Africa; SAE134_08, FJ464380, South Africa; SA381_00, EF429199, South Africa; SA93_01, EF429198, South Africa; SPU116_89, EF429197, South Africa; goshawk_Hungary_04, DQ116961, Hungary; B956 polyprotein gene-1937, AY532665, Uganda; B956 117B3, M12294, Uganda; ArD76104, DQ318019, Senegal; H442, EF429200, South Africa; ArB3573_82, DQ318020, Central African Republic; Sarafend, AY688948, Uganda; Madagascar AnMg798, DQ176636, Madagascar; PTRoxo, AM404308, Portugal; Egypt101, AF260968, Egypt; EthAn4766, AY603654, Ethiopia; Kunjin MRM61C, D00256, Australia; WNV Italy 1998 equine, AF404757, Italy; WNV Ast02–2-25, DQ374653, Russia; LEIV-Vlg00–27924, AY278442, Russia; LEIV-Krnd88–190, AY277251, Russia; goose_Hungary_03, DQ118127, Hungary; NY385_99, DQ211652 NY, USA; NY99-eqhs, AF260967 NY, USA; NY99-flamingo382–99, AF196835 NY, USA; TYP9376 NY385_99, AY848697 NY, USA; WNV TX 2002 02, DQ164206 TX, USA; TM171_03, AY371271, Mexico; Mex03, AY660002, Mexico; TX 2002-HC, DQ176637 TX, USA; WNV804994, DQ256376, India; Rabensburg 97–103, AY765264, Czech Republic; JEV JaOAr5982, M18370, Japan.

**Figure 3:**
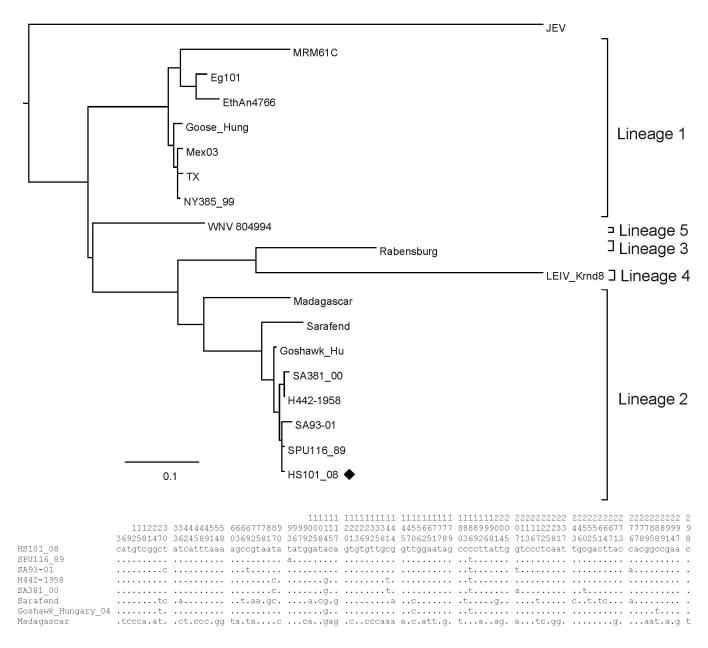
Maximum-likelihood analysis of the E-protein region of a West Nile virus isolate, HS101_08 (black diamond), recovered from horses in 2008 compared with isolates obtained from humans and animals from South Africa and other regions of the world. Nucleotide differences between lineage 2 strains included in the alignment are shown in the summarized alignment below the tree, indicating only unique nucleotides. Vertical numbers above the alignment indicate the position of each variable site on the gene fragment. Scale bar indicates nucleotide substitutions per site.

The 5 specimens that were positive for WNV by RT-PCR were subjected to sequence analysis and confirmed to be WNV by BLAST (www.ncbi.nlm.nih.gov/blast/Blast.cgi) search analysis. Phylogenetic comparison of the 189-bp NS5 gene region with representative sequences of all 5 currently recognized WNV lineages confirmed that all strains clustered with lineage 2 (Figure 2) and were closely related to lineage 2 strains isolated from humans in South Africa. The horse specimens differed by 0%–3% nucleotides from South African lineage 2 strains in the NS5 region. Recent (2008) strains displayed the least variation from each other (0%–1.2%) and were most closely related to strains SPU116/89 and SA93/01, which had been isolated from a person with fatal hepatitis and nonfatal encephalitis, differing by 1.2% and 1.8%, respectively. Specimens from southern Africa had 1.2%–4.8% differences compared with the lineage 2 Hungary isolate of 2004. All lineage 2 strains from southern Africa differed by 18.6%–19.2% from the Madagascar strain and by 19.2%–25.7% with lineage 1 strains. Additional phylogenetic analysis of a more variable region of the E-protein was performed on the 2008 isolate obtained from the brain of HS101/08 (Figure 3). HS101/08 had 0.07%–2% differences in the E-protein region compared with all other southern Africa lineage 2 strains and was the closest to SPU116/89; it differed by 6.9% from the prototype Uganda strain (B956), differed by 17.3% from a strain from Madagascar, and 23% from NY385/99 (lineage 1). The 2004 Hungary lineage 2 strains differed by only 1.6% from the southern Africa strains, which suggests that the Hungary strains may have originated from southern Africa. On the amino acid level, all southern Africa lineage 2 strains’ E-protein regions were identical to each other and to the Hungary strain but differed by 7% from the Madagascar strain and by 23% from the lineage 1 NY385/99 strain.

## Discussion

Lineage 2 WNV is known to be endemic to southern Africa; however, few cases of WNV disease have been reported in recent years and the role of lineage 2 as a human and horse pathogen has been disputed (*3*,*13*). Our previous investigations of the pathogenicity of lineage 2 strains in humans and mice have indicated the existence of lineage 2 strains that are highly pathogenic and neuroinvasive in mice (*2*,*20*). WNV is rarely considered in a differential diagnosis for neurologic disease of humans or horses in southern Africa. In the Northern Hemisphere, horses are highly susceptible and develop severe WNV disease and thus have been used as sentinels for human cases (*35*). Most indigenous birds in southern Africa do not display disease despite a high seroprevalence for WNV infection (*17*), which suggests that genetic resistance may exist in local birds.

We investigated 80 cases of unexplained disease in horses compatible with WNV clinical signs. It can be expected that not all cases were identified but rather that samples from only economically valuable horses were sent in for laboratory investigation when unexplained neurologic signs and fevers were noted by the resident veterinarian. For 7 horses, WNV could be confirmed by RT-PCR and virus isolation (*5*) or as the probable cause of signs due to the presence of IgM confirmed by neutralization assays, suggesting a recent WNV infection. All 7 horses had substantial neurologic signs. The fact that 5 cases were fatal suggests that up to 7 (21.8%) of 32 cases of undetermined neurologic disease investigated in horses in South Africa over 16 months were caused by WNV. The high mortality rate (5 [71%] of 7 horses) and the clinical signs correlated with findings of neurologic disease in horses in the Northern Hemisphere.

Samples from 5 horses were positive by nested real-time RT-PCR (4 brain tissue specimens and 1 serum sample), and 2 were IgM positive. These findings indicate that these were useful diagnostic and surveillance tools.

Sequencing of cDNA confirmed that all WNV infections were caused by lineage 2 and were closely related to lineage 2 strains previously isolated from human case-patients in South Africa. The NS5 gene regions of the 2008 strains were most closely related to each other and to recent human isolates. The WNV SPU93/01 isolate was recovered from an immunocompetent adult who was hospitalized with encephalitis in Johannesburg in 2001; the SPU116/89 isolate was recovered from the liver of a human patient with fatal hepatitis. Analysis of horse isolate HS101/08 indicated that the 2008 strains were unique in the E-protein region and most closely related to SPU116/89, followed by SPU93/01. Each of these strains is highly neuroinvasive in mice (*18*,*20*).

All cases were identified in late summer to autumn, timing that coincides with AHSV and EEV outbreaks in South Africa (*36*,*37*). The extent of these cases caused by concurrent and cocirculating viruses may contribute to the underrecognition of WNV cases in horses in southern Africa. Some of the WNV cases reported here were submitted as suspected AHSV and EEV infections. AHSV is an insect-borne orbivirus that causes a noncontagious disease of equids and is associated with high death rates in sub-Saharan Africa. African horse sickness occurs in 4 forms: horse sickness fever (mild), cardiac (>50% mortality rate), mixed (75% mortality rate), and pulmonary (95% mortality rate). Signs of the cardiac form include subcutaneous edema, particularly of the head, neck, chest, and supraorbital fossae. The pulmonary form is peracute and may develop so rapidly that an animal can die without previous signs of illness. It is characterized by depression, fever, respiratory distress, severe dyspnea and coughing spasms, and severe sweating; terminally, quantities of frothy fluid may be discharged from the nares and periods of recumbency may occur (*37*). Annual vaccination with a live attenuated polyvalent AHSV vaccine provides protection against most of the 9 serotypes, although vaccine failures have been reported and outbreaks continue to occur. Vaccination complicates diagnosis by serologic testing, and virus may be isolated from horses after vaccination (*38*). EEV is a closely related orbivirus also widespread in southern Africa; it may cause fever, abortion, and neurologic involvement (*39*). Outbreaks of both occur annually and are associated with unusually high rainfall and increased vector (*Culicoides* midges) populations (*40*), which will also favor an increase in *Culex* mosquitoes, which transmit WNV. The 2007–2008 summer and late autumn seasons in South Africa were marked by unusually high rainfall, and the numbers of AHSV and EEV cases were also high (www.africanhorsesickness.co.za). Clinical signs of AHSV do not resemble those of WNV, and neurologic signs are not characteristic; but in the absence of laboratory testing, horses that die of unexplained causes may be dismissed as having been infected with AHSV. The identification of 2 AHSV–WNV co-infections in this study is therefore not surprising. In both cases, veterinarians and owners noted neurologic signs, which are not typical of AHSV. These co-infections may increase the disease severity for each virus and should be taken into consideration in areas where both diseases are endemic. In most (5/7) WNV cases identified in this study, no other pathogens were identified. All identified horses with WNV infection had neurologic involvement, which could be used to distinguish WNV–AHSV co-infections from conventional AHSV infections. None of the identified horses with EEV had neurologic signs, although EEV virus was isolated from several horses with fever.

Our findings should raise awareness that WNV lineage 2 can cause neurologic disease in both horses and humans in southern Africa. In the absence of bird deaths, detection of cases in horses may serve as an early warning system for WNV outbreaks among humans.

## References

[1] Hayes EB, Sejvar JJ, Zaki SR, Lanciotti RS, Bode AV, Campbell GL. Virology, pathology, and clinical manifestations of West Nile virus disease. Emerg Infect Dis. 2005;11:1174–9. 16102303 10.3201/eid1108.050289b PMC3320472

[2] Burt FJ, Grobbelaar AA, Leman PA, Anthony FS, Gibson GV, Swanepoel R. Phylogenetic relationships of southern African West Nile virus isolates. Emerg Infect Dis. 2002;8:820–6. 12141968 10.3201/eid0808.020027 PMC2732512

[3] Lanciotti RS, Roehrig JT, Deubel V, Smith J, Parker M, Steele K, Origin of the West Nile virus responsible for an outbreak of encephalitis in the northeastern United States. Science. 1999;286:2333–7. 10.1126/science.286.5448.2333 10600742

[4] Lvov DK, Butenko AM, Gromashevsky VL, Kovtunov AI, Prilipov AG, Kinney R, West Nile virus and other zoonotic viruses in Russia: examples of emerging-reemerging situations. Arch Virol Suppl. 2004;18:85–96. 15119764 10.1007/978-3-7091-0572-6_7

[5] Bakonyi T, Hubalek Z, Rudolf I, Nowotny N. Novel flavivirus or new lineage of West Nile virus, central Europe. Emerg Infect Dis. 2005;11:225–31. 15752439 10.3201/eid1102.041028 PMC3320449

[6] Bondre VP, Jadi RS, Mishra AC, Yergolkar PN, Arankalle VA. West Nile virus isolates from India: evidence for a distinct genetic lineage. J Gen Virol. 2007;88:875–84. 10.1099/vir.0.82403-0 17325360

[7] Campbell GL, Marfin AA, Lanciotti RS, Gubler DJ. West Nile virus. Lancet Infect Dis. 2002;2:519–29. 10.1016/S1473-3099(02)00368-7 12206968

[8] Petersen LR, Marfin AA. West Nile virus: a primer for the clinician. Ann Intern Med. 2002;137:173–9. 12160365 10.7326/0003-4819-137-3-200208060-00009

[9] Ward MP, Levy M, Thacker HL, Ash M, Norman SK, Moore GE, Investigation of an outbreak of encephalomyelitis caused by West Nile virus in 136 horses. J Am Vet Med Assoc. 2004;225:84–9. 10.2460/javma.2004.225.84 15239478

[10] Schuler LA, Khaitsa ML, Dyer NW, Stoltenow CL. Evaluation of an outbreak of West Nile virus infection in horses: 569 cases (2002). J Am Vet Med Assoc. 2004;225:1084–9. 10.2460/javma.2004.225.1084 15515988

[11] Dauphin G, Zientara S, Zeller H, Murgue B. West Nile: worldwide current situation in animals and humans. Comp Immunol Microbiol Infect Dis. 2004;27:343–55. 10.1016/j.cimid.2004.03.009 15225984

[12] Nielsen CF, Reisen WK, Armijos MV, Maclachlan NJ, Scott TW. High subclinical West Nile virus incidence among nonvaccinated horses in northern California associated with low vector abundance and infection. Am J Trop Med Hyg. 2008;78:45–52. 18187784

[13] Guthrie AJ, Howell PG, Gardner IA, Swanepoel RE, Nurton JP, Harper CK, West Nile virus infection of thoroughbred horses in South Africa (2000–2001). Equine Vet J. 2003;35:601–5. 10.2746/042516403775467180 14515962

[14] Ward MP, Schuermann JA, Highfield LD, Murray KO. Characteristics of an outbreak of West Nile virus encephalomyelitis in a previously uninfected population of horses. Vet Microbiol. 2006;118:255–9. 10.1016/j.vetmic.2006.07.016 16971067

[15] Dauphin G, Zientara S. West Nile virus: recent trends in diagnosis and vaccine development. Vaccine. 2007;25:5563–76. 10.1016/j.vaccine.2006.12.005 17292514

[16] Beasley DW. Recent advances in the molecular biology of West Nile virus. Curr Mol Med. 2005;5:835–50. 10.2174/156652405774962272 16375717

[17] Jupp PG. The ecology of West Nile virus in South Africa and the occurrence of outbreaks in humans. Ann N Y Acad Sci. 2001;951:143–52. 11797772 10.1111/j.1749-6632.2001.tb02692.x

[18] Botha EM, Markotter W, Wolfaardt M, Paweska JT, Swanepoel R, Palacios G, Genetic determinants of virulence in pathogenic lineage 2 West Nile virus strains. Emerg Infect Dis. 2008;14:222–30. 10.3201/eid1401.070457 18258114 PMC2600181

[19] Bakonyi T, Ivanics E, Erdelyi K, Ursu K, Ferenczi E, Weissenbock H, Lineage 1 and 2 strains of encephalitic West Nile virus, central Europe. Emerg Infect Dis. 2006;12:618–23. 16704810 10.3201/eid1204.051379 PMC3294705

[20] Venter M, Myers TG, Wilson MA, Kindt TJ, Paweska JT, Burt FJ, Gene expression in mice infected with West Nile virus strains of different neurovirulence. Virology. 2005;342:119–40. 10.1016/j.virol.2005.07.013 16125213

[21] Bunning ML, Bowen RA, Cropp CB, Sullivan KG, Davis BS, Komar N, Experimental infection of horses with West Nile virus. Emerg Infect Dis. 2002;8:380–6. 11971771 10.3201/eid0804.010239 PMC3393377

[22] Beasley DW, Davis CT, Whiteman M, Granwehr B, Kinney RM, Barrett AD. Molecular determinants of virulence of West Nile virus in North America. Arch Virol Suppl. 2004;18:35–41. 15119761 10.1007/978-3-7091-0572-6_4

[23] Zaayman D, Human S, Venter M. A highly sensitive method for the detection and genotyping of West Nile virus by real-time PCR. [**PMID: 19138708**]. J Virol Methods. 2009;157:155–60. 10.1016/j.jviromet.2008.12.014 19138708

[24] Berthet FX, Zeller HG, Drouet MT, Rauzier J, Digoutte JP, Deubel V. Extensive nucleotide changes and deletions within the envelope glycoprotein gene of Euro-African West Nile viruses. J Gen Virol. 1997;78:2293–7. 9292017 10.1099/0022-1317-78-9-2293

[25] Guindon S, Gascuel O. A simple, fast, and accurate algorithm to estimate large phylogenies by maximum likelihood. Syst Biol. 2003;52:696–704. 10.1080/10635150390235520 14530136

[26] Tamura K, Dudley J, Nei M, Kumar S. MEGA4: Molecular Evolutionary Genetics Analysis (MEGA) software version 4.0. Mol Biol Evol. 2007;24:1596–9. 10.1093/molbev/msm092 17488738

[27] Swanepoel R, Struthers JK, Erasmus MJ, Shepherd SP, McGillivray GM, Erasmus BJ, Comparison of techniques for demonstrating antibodies to Rift Valley fever virus. J Hyg (Lond). 1986;97:317–29. 3537118 10.1017/s0022172400065414 PMC2083537

[28] Paweska JT, Burt FJ, Swanepoel R. Validation of IgG-sandwich and IgM-capture ELISA for the detection of antibody to Rift Valley fever virus in humans. J Virol Methods. 2005;124:173–81. 10.1016/j.jviromet.2004.11.020 15664066

[29] Paweska JT, Burt FJ, Anthony F, Smith SJ, Grobbelaar AA, Croft JE, IgG-sandwich and IgM-capture enzyme-linked immunosorbent assay for the detection of antibody to Rift Valley fever virus in domestic ruminants. J Virol Methods. 2003;113:103–12. 10.1016/S0166-0934(03)00228-3 14553896

[30] Niedrig M, Sonnenberg K, Steinhagen K, Paweska JT. Comparison of ELISA and immunoassays for measurement of IgG and IgM antibody to West Nile virus in human sera against virus neutralisation. J Virol Methods. 2007;139:103–5. 10.1016/j.jviromet.2006.09.009 17084464

[31] House C, Mikiciuk PE, Berninger ML. Laboratory diagnosis of African horse sickness: comparison of serological techniques and evaluation of storage methods of samples for virus isolation. J Vet Diagn Invest. 1990;2:44–50. 2128615 10.1177/104063879000200108

[32] Bremer CW, Viljoen GJ. Detection of African horsesickness virus and discrimination between two equine orbivirus serogroups by reverse transcription polymerase chain reaction. Onderstepoort J Vet Res. 1998;65:1–8. 9629584

[33] Goldwasser RA, Kissling RE. Fluorescent antibody staining of street and fixed rabies virus antigens. Proc Soc Exp Biol Med. 1958;98:219–23. 13554598 10.3181/00379727-98-23996

[34] Haines DM, Chelack BJ. Technical considerations for developing enzyme immunohistochemical staining procedures on formalin-fixed paraffin-embedded tissues for diagnostic pathology. J Vet Diagn Invest. 1991;3:101–12. 2039784 10.1177/104063879100300128

[35] Ward MP, Scheurmann JA. The relationship between equine and human West Nile virus disease occurrence. Vet Microbiol. 2008;129:378–83. 10.1016/j.vetmic.2007.11.022 18182255

[36] Quan M, van Vuuren M, Howell PG, Groenewald D, Guthrie AJ. Molecular epidemiology of the African horse sickness virus S10 gene. J Gen Virol. 2008;89:1159–68. 10.1099/vir.0.83502-0 18420793

[37] Mellor PS, Hamblin C. African horse sickness. Vet Res. 2004;35:445–66. 10.1051/vetres:2004021 15236676

[38] Von Teichman BF, Smit TK. Evaluation of the pathogenicity of African horsesickness (AHS) isolates in vaccinated animals. Vaccine. 2008;26:5014–21. 10.1016/j.vaccine.2008.07.037 18682269

[39] Erasmus BJ, Adelaar TF, Smit JD, Lecatsas G, Toms T. The isolation and characterization of equine encephalosis virus. Bull Off Int Epizoot. 1970;74:781–9.

[40] Meiswinkel R. The 1996 outbreak of African horse sickness in South Africa—the entomological perspective. Arch Virol Suppl. 1998;14:69–83. 9785497 10.1007/978-3-7091-6823-3_8

